# Effect of Nutritive and Non-Nutritive Sweeteners on the Lipid Profile, Castelli Index I and II, and Atherogenic Index of Plasma Using Experimental Rat Models

**DOI:** 10.1155/jnme/8602969

**Published:** 2025-05-27

**Authors:** Ruth T. Owu, Efua E. Annan, Joana Ainuson-Quampah, Matilda Asante, Charles Addoquaye Brown, George A. Asare

**Affiliations:** ^1^Department of Dietetics, University of Ghana, Accra, Ghana; ^2^Department of Medical Laboratory Sciences, University of Ghana, Accra, Ghana

## Abstract

Previous research on sweeteners' effect on health has focused on indices of cardiometabolic risk factors without considering lipid ratios such as the Atherogenic Index of Plasma (AIP) and Castelli Risk Index I and II (CRI-I and CRI-II). The study sought to evaluate the effect of natural sweeteners on lipid profiles and lipid ratios. Seventy-eight female Sprague Dawley rats (6 rats per group) were administered with different doses of sweeteners (3 groups per sweetener): white sugar (0.035 g/mL, 0.07 g/mL, and 0.1 g/mL), brown sugar (0.036 g/mL, 0.072 g/mL, and 0.11 g/mL), honey (0.047 g/mL, 0.094 g/mL, and 0.14 g/mL) and stevia (0.004 g/mL, 0.014 g/mL, and 0.021 g/mL) for 17 weeks. The highest weight gain was observed with high-dose stevia administration (72.7 g ± 10.5). The group administered with high dose of white sugar had the highest CRI-I (1.79 ± 0.11) and CRI-II (0.49 ± 0.09). CRI-I and CRI-II had a dose-dependent increase with white sugar. The AIP was highest in the high-dose stevia group (0.21 ± 0.07) with dose-dependent increases within the stevia group. High intakes of white sugar and stevia tend to promote the development or progression of atherosclerosis.

## 1. Introduction

Cardiovascular disease (CVD) is a term used to refer to diseases of the heart and blood vessels—the veins and arteries. According to the World Health Organisation (WHO), CVD remains the leading cause of morbidity and mortality globally, representing 32% of global mortality (17.9 million) with the majority of the deaths (< 10 million) occurring in lower- to middle-income countries [1, 2].

Behavioural risk factors associated with CVD include physical inactivity, smoking, alcohol intake and intake of unhealthy diet which includes intake of high-caloric foods, saturated fats and sugars [3]. Unhealthy dietary intake increases CVD risk through its effect on body weight, lipid profile, blood pressure, inflammation and endothelial function [4]. The prevalence of CVDs and metabolic syndrome is increasing statistically and chronologically with an increase in the intake of sweeteners [5].

Sweeteners can be categorised into two [2] groups based on their caloric contents: nutritive sweeteners (NS) which contain carbohydrates and provide approximately 4 kcal/g and low or no calorie sweeteners (LNCS) providing minimal or no calories (LNCS) [6]. Commonly consumed NS added to food including honey, white granulated sugar, brown sugar, molasses, and corn syrup [7]. There are currently eight [8] LNCS approved by the United States Food and Drug Administration namely acesulfame-K (ACK), aspartame, neotame, saccharin, sucralose, stevia, advantame and Luo Han Guo fruit extract [8] with 11 approved by the European Union: ACK, aspartame, cyclamate, saccharin, sucralose, thaumatin, neohesperidine, steviol glycosides, neotame, aspartame-acesulfame salt and advantame [9]. NS either occur naturally in foods or are added during food processing or before food consumption by consumers, whereas LNCS are primarily used as tabletop packets, diet soft drinks and products such as bread, cereals, granola bars, sugar-free yoghurts or ice-cream, condiments, sugar-free jam, medications, multivitamins, flavoured toothpaste and mouthwash [6, 10].

Dietary sugars have been associated with hyperlipidaemia resulting in arteriosclerosis—an accumulation and deposition of cholesterol and fatty deposits in the walls of the arteries [11]. Major indicators that are frequently used to predict atherosclerosis include a lipid profile that evaluates total cholesterol (TC), high-density lipoprotein (HDL) cholesterol, low-density lipoprotein (LDL) cholesterol and triglycerides (TGs) [12]. However, studies have shown that lipid ratios such as the Atherogenic Index of Plasma (AIP), Castelli Risk Index I and II (CRI I and II), atherogenic coefficient (AC), and non-high-density lipoprotein cholesterol (NHC) are diagnostic alternatives used in predicting the risk of developing atherosclerosis when the conventional lipid parameters (TG, HDL C, LDL C and TC) remain ostensibly normal [13].

Previous research on the effect of sweeteners on health has focused on indices such as glucose, insulin signalling, inflammation, anthropometric measurements, microbiota and lipid profile without considering lipid ratios such as the atherogenic index [14–16]. The study aimed to examine the effect of different types and doses of NS (white sugar, brown sugar and honey) and LCNS (stevia sweetener) on the CRI I and II and the AIP. An experimental animal model involving rats (Sprague Dawley rats) was used in this study. Rats are good models for studying metabolic conditions that mimic what is seen in humans [17]. Sprague Dawley rats were selected due to their availability, ease of handling and suitability for comparison with previous studies.

## 2. Materials and Methods

### 2.1. Experimental Procedure

A true experimental study was carried out at the Department of Animal Experimentation at the Noguchi Memorial Institute for Medical Research (NMIMR) from February–May, 2023. All animal procedures and techniques used in this study were in accordance with the National Institute of Health Guidelines for the Care and Use of Laboratory Animals [18]. The research protocol was approved by the Institutional Animal Care and Use Committee of the Noguchi Memorial Institute of Medical Research (UG-IACUC 001/21-22).

Seventy-eight (78) adult female Sprague Dawley rats, weighing between 130 g and 200 g, were obtained from the Department of Animal Experimentation at the NMIMR. Female rats were selected based on OECD recommendations for acute toxicity studies [19, 20]. While the OECD Test Guideline 452 outlines the design of chronic toxicity studies in rodents and recommends the use of both sexes, it allows for the use of a single sex when scientifically justified. The choice of female rats was based on their distinct metabolic profiles such as differences in glucose tolerance, insulin sensitivity and lipid metabolism which are central to the study of metabolic syndrome [21, 22]. Furthermore, female Sprague Dawley rats have been underrepresented in preclinical metabolic studies involving sweetener intake. Their inclusion in this study promotes more inclusive and translational findings.

The rats were housed in propylene cages (43 × 27 × 15 cm) at the Department of Animal Experimentation at the NMIMR under standard temperature conditions (24°C ± 2°C) and relative humidity (60%–70%) with a 12 h light/dark cycle ensured. The animals were monitored daily for their health status. No adverse events were observed during and at the end of the study. Rat chow was formulated and supplied by AGRIFEEDS, an agri-feed enterprise in Ghana. The experimental animals were acclimatized for 2 weeks before the commencement of the study. The rats were randomly split into experimental groups according to the sweetener type and dosage: control (C), white sugar low dose (WSLD), white sugar medium dose (WSMD), white sugar high dose (WSHD), brown sugar low dose (BSLD), brown sugar medium dose (BSMD), brown sugar high dose (BSHD), honey low dose (HLD), honey medium dose (HMD), honey high dose (HHD), stevia low dose (SLD), stevia medium dose (SMD), and stevia high dose (SHD) (*n* = 6/group) (Table 1). Baseline fasting blood glucose (FBG) and body weight were measured.

### 2.2. Preparation of Sweetener Solution

The experimental groups with their dosages are shown in Table 2. The percentage of sugar solutions for NS was calculated according to the WHO recommendation for reducing the intake of free sugars to less than 10% of total energy intake and a further reduction of the intake of free sugars to below 5% of total energy intake [23]. For each of the NS, 5% was used for the low dose, 10% for the medium dose and 15% for the high dose. The dosages were adjusted weekly based on the weight of the animals. The sweeteners were administered orally by gavage for 17 weeks every morning. The gavage technique was used to ensure complete delivery of the sweeteners. The brown and white sugar (sucrose) were purchased from OXY industries (Tema, Ghana) (suppliers of sunny brown and white sugar). The stevia sweetener (stevia extract (Rebiana) and maltodextrin) was purchased from Walmart Canada, and the honey was purchased from the Centre for Scientific Institute for Medical Research, Accra Ghana.

### 2.3. Euthanisation and Collection of Blood Serum

At the end of 17 weeks, the rats were fasted overnight (16 h) and anesthetized 0.1 mL/100 g of body weight of Anaket and Chinazin (4:1). Blood was then sampled by cardiac puncture. Using a 5-mL syringe with a 23 G needle, 5 mL of blood was drawn and transferred into lithium heparin tubes which were subsequently centrifuged at 1008 RCF for 10 min to separate the serum. The sera were transferred into sera separator tubes and preserved at 4°C until further analysis. The stored samples were allowed to thaw to room temperature of 25°C before assaying.

### 2.4. Measurement of Food Intake and Body Weight

The animals were allowed access to drinking water ad libitum. Food intake was measured daily throughout the study. Total caloric intake was determined at the end of the experiment by multiplying the amount of food consumed by the caloric contents per gram of chow as stated by the manufacturer (2.89 kcal/g) [24]. The rats were weighed weekly at the same time using an animal scale. Per cent body weight gain was calculated as shown:

(1) $$\begin{array}{c} \text{Percent weight change}=\frac{\text{endline weight}‐\text{initial weight}}{\text{initial weight}}\times 100. \end{array}$$

The body mass index and the index lees index were calculated according to the method by Samat et al. [25].

### 2.5. Biochemical Parameters

Plasma lipid profiles consisting of TC, TG, HDL and LDL were analysed with an auto-analyser machine and test kits according to the manufacturer's instructions (Mindray Bio-Medical Electronics Bs-200e, Shenzhen, China). Castelli Index I and II were estimated as TC/HDL and LDL/HDL, respectively [26]. The AIP was calculated as log(TG/HDL) [27].

### 2.6. Statistical Analysis

Statistical analysis was performed using GraphPad Prism 9.5.1. Normality was assessed using the Kolmogorov–Smirnov test. The effect of the sweeteners on the lipid profile, lipid ratios and percent weight gain was assessed using one-way ANOVA with Tukey's post hoc test conducted where differences were statistically significant. Statistical significance was set at *p* < 0.05. Results are presented as mean ± SEM.

## 3. Results

There were no treatment-related deaths throughout the 17 weeks of the study. No apparent differences in physical activity or behaviours, no significant changes in stool, urine, eye colour, diarrhoea, salivation, convulsion, sleep or comma and no significant loss of fur or skin lesions were observed.

### 3.1. Effect of the Intake of Sweeteners on Energy Intake and Body Weight Changes

**Figure 1:**
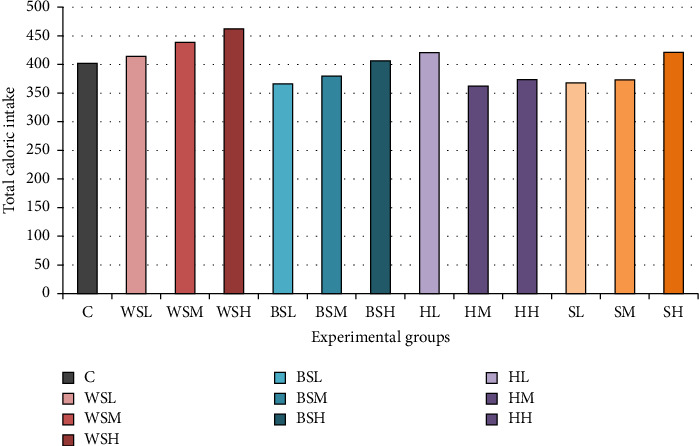
Energy intake after 17 weeks of treatment. C = control, WSL = white sugar low, WSM = white sugar medium, WSH = white sugar high, BSL = brown sugar low, BSM = brown sugar medium, BSH = brown sugar high, HL = honey low, HM = honey medium, HH = honey high, SL = stevia low, SM = stevia medium, and SH = stevia high.

**Figure 2:**
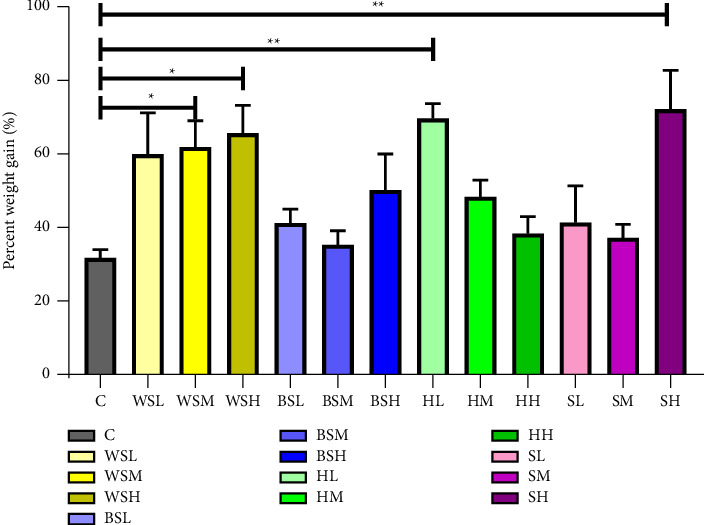
Effect of sweeteners on percentage weight change after 17 weeks of treatment. C = control, WSL = white sugar low, WSM = white sugar medium, WSH = white sugar high, BSL = brown sugar low, BSM = brown sugar medium, BSH = brown sugar high, HL = honey low, HM = honey medium, HH = honey high, SL = stevia low, SM = stevia medium, and SH = stevia high. ^∗^*p* < 0.05, ^∗∗^*p* < 0.01.

The effect of the intake of NS and non-NS on energy intake and percentage body weight gain of the rats measured over the period of 17 weeks is shown in Figures 1 and 2, respectively. The group fed with high-dose white sugar had the highest caloric intake (462 kcal) and the lowest intake in medium dose honey (362 kcal). Increasing sweetener dosages paralleled an increase in energy intake for white sugar, brown sugar and stevia groups (Figure 1).

The average percent weight gain in all the treatment groups was observed to be higher than the control group with high-dose stevia recording the highest weight gain (72.7%). An increase in the dosages of the various sweeteners was observed to parallel an increase in the percentage of body weight gain except for the groups consuming honey. Percent weight gain was statistically significantly different between different sweetener groups and dosages (*F* (12, 65) = 3.953, *p* < 0.001).

### 3.2. Effect of the Intake of Sweeteners on the Lipid Profile and Lipid Ratios

#### 3.2.1. Total Cholesterol

**Figure 3:**
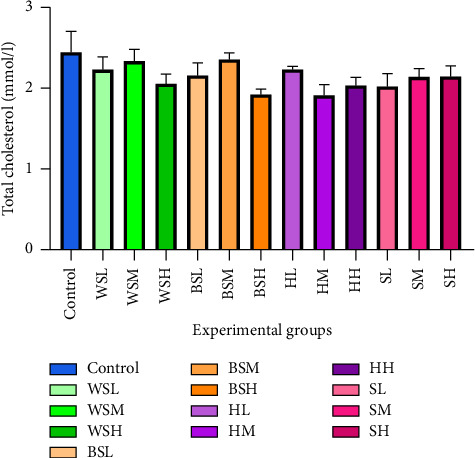
Total cholesterol after 17 weeks of treatment. C = control, WSL = white sugar low, WSM = white sugar medium, WSH = white sugar high, BSL = brown sugar low, BSM = brown sugar medium, BSH = brown sugar high, HL = honey low, HM = honey medium, HH = honey high, SL = stevia low, SM = stevia medium, and SH = stevia high.

It is evident that there were no significant differences in the TC between the various treatment groups (*p* = 0.267) (Figure 3).

#### 3.2.2. TG

The TG level was lowest in the brown sugar low dose (0.67 ± 0.08 mmol/L) and highest in rats administered with high-dose stevia (2.66 ± 0.37 mmol/L) (Table 3). The groups administered with stevia had TG levels increasing in a dose-dependent manner. An ANOVA test showed a statistically significant difference in the mean TG concentration among the various treatment groups [*F* (12.0, 65.0) = 2.9, *p* = 0.003].

Within each sweetener group, only honey showed significant differences in TG concentration, when comparing the different doses of honey. Low-dose honey had a lower TG concentration than medium and high-dose honey. Also comparing the various dosage groups, all dosages of stevia had higher TG concentration compared with the other sweeteners with the same dosage levels.

#### 3.2.3. HDL

With the evidence of the effect of the different experimental groups on HDL, the group administered with low-dose honey had the highest HDL concentration (1.72 ± 0.19 mmol/L) (Table 4). There was a statistically significant difference among the various treatment groups (*F* (12, 65) = 2.0, *p* = 0.038). The greatest reduction in HDL was seen in high-dose white sugar administration (Table 4).

#### 3.2.4. LDL

In comparing the effect of treatment type on LDL-c, the experimental group administered with medium-dose white sugar had the highest LDL (0.55 ± 0.07 mmol/L) (Table 5). The results as shown in Table 5 indicate that there was a statistically significant difference in LDL-c among the various study groups. In pulling the medium dosage groups together, white sugar had a higher LDL concentration than brown sugar, honey and stevia (Table 5).

#### 3.2.5. Very LDL

The highest concentration of VLDL was in the group administered with high-dose brown sugar (0.68 ± 0.24 mmol/L) (Table 6). Generally, VLDL-c was low with the intake of white sugar and highest with the intake of brown sugar.

### 3.3. CRI I

The effect of varying treatments on CRI I is reported in Table 7. The group administered with high-dose white sugar had the highest CRI-I score (2.30 ± 0.3). A significant association was found between the experimental groups [*F* (12, 65) = 4.5, *p* < 0.0001], specifically within the high dose group, and white sugar had a significantly higher CRI-I than brown sugar, honey and stevia (Table 7). Thus, administration of high-dose white sugar was observed to increase CRI I.

### 3.4. CRI II

CRI II was highest with high dose of white sugar (0.49 ± 0.09) (Table 8). CRI-II increased in a dose-dependent manner for white sugar. The greatest effect on CRI-II was observed with white sugar administration (Table 8).

### 3.5. AIP

The highest AIP was observed in high-dose stevia and lowest in the group administered with low-dose honey (Table 9). ANOVA showed a significant association between treatment groups and the AIP (*p* < 0.0001). AIP increase in the stevia group was in a dose-dependent manner. Generally, stevia was observed to cause an increase in the AIP (Table 9).

## 4. Discussion

The health effects related to the intake of nutritive and non-NS are complex and remain a topic of debate. Each sweetener used in this study contains specific compounds that may elicit varied physiological responses beyond caloric contribution. For instance, stevia sweeteners contain steviol glycosides and maltodextrin (in the commercial formulation used), which may have additional biological effects. Percent weight gain was observed across all the experimental groups over the study period. Several studies using large numbers do suggest that artificial sweeteners do cause weight gain. However, the NSS evaluated in those studies was mostly found in beverages. Evidence suggests that intake of high intensity sweeteners may increase consumption of sweet flavours and increase in appetite, resulting in increased food intake, hence promoting weight gain [28]. Although stevia extract is generally recognized as safe, commercial stevia products especially those combined with carriers such as maltodextrin have been linked to weight gain in some studies. Similar to other LNCS, stevia may activate sweet taste receptors and trigger hyperinsulinemia, leading to increased fat storage and a heightened risk of insulin resistance. Additionally, because LNCS are not digested in the upper gastrointestinal tract, they reach the colon intact where they interact with gut microbiota. This interaction can induce gut dysbiosis, altering microbial fermentation and producing metabolites associated with insulin resistance and metabolic dysfunction. Another proposed mechanism involves the reduction of satiety signals, which may encourage excess energy intake and further contribute to weight gain. [29]. In this study, the high-dose stevia group had an increased energy intake coupled with an increase in its food efficiency ratio. The body mass index on the other hand did not show any difference among the various groups in this study.

Surprisingly, honey had an inverse relationship, where the highest dose within the group caused the least weight gain. Weight gain within the honey group was dose-dependent. We do question if this phenomenon is due to the benefits of the antioxidants in honey as against the sugars (Figure 2). It has been postulated that the active components in honey, flavonoids and phenols have antiobesity effects by preventing the formation of fatty acids and triglycerol and enhancing the breakdown of fat [30]. Similarly, acute administration of honey in both female and male Sprague Dawley rats was reported to induce weight loss [25, 31]. Similarities in these findings may support the evidence that honey possesses weight-reducing properties. However, the findings by Atangwho et al. [32] observed increased weight gain in female Wistar rats fed with high dose of white sugar and high dose honey compared to the male Wistar rats. Interestingly, the highest weight increase was observed with stevia, and the dose was statistically significant compared to the control (*p* = 0.043).

Within every group of this study, low doses of sweeteners had less effect on TG within the group. Low-dose brown sugar had a better TG value than all the groups. Stevia levels were generally higher than other sugar groups. Furthermore, it was the only group in which increases were in a dose-dependent manner with the highest dose being 2.66 mmol/L compared to the control 0.83 mmol/L, almost 3.5x that of the control (*p* = 0.001). Low-dose brown sugar was the best TG lowing agent. Stevia, on the other hand, had the worst effect on TG. All doses of stevia were significantly different from the control (< 0.000, < 0.000 and 0.012, respectively). In a meta-analysis of 14 randomized controlled trials of NNS including stevia on the lipid profile, it was suggested that NNS did not have any effect on TG, TC, LDL and HDL. However, further analysis of subgroups revealed that NNS could be related to increasing in LDL. Although the increase was small, it was statistically significant [33]. In a study with NNS, high TG levels were observed in rats on high doses. Similarly, LDL was elevated in rats on high doses as well as those on low doses. When treatment was withdrawn after 12 weeks of substance administration, a sharp drop occurred in LDL levels of rats fed aspartame. On the contrary, HDL increased slightly but not significantly. However, pathological changes in the liver and kidney were not recovered [34].

In this study, the effect of chronic intake of sweeteners on HDL-c was observed in the group treated with high-dose white sugar. HDL-c was significantly low in the high-dose white sugar group. HDL-c is referred to as the good cholesterol, known to be protective against heart disease by eliminating bad cholesterol from the blood which is a major risk factor for CVD [35]. High intake of sucrose has been associated with metabolic conditions such as diabetes, CVD and obesity. One of its plausible mechanisms may be its effect on TG and HDL-c concentration which can facilitate atherogenicity [36, 37].

In this study, LDL cholesterol was lower in all groups compared to the control except for the WSMD, whereas the lowest values were in the HMD, stevia low dose and BSHD groups. Differences were statistically significant (0.010 and 0.026, respectively). In a 14-day study, honey reduced LDL levels significantly (alongside TC and TG) in Wistar rats fed a high-fat diet [38]. Significant decreases were also observed between the control and stevia low and high doses. In a study involving 60 healthy subjects (18–30 years) randomly assigned to two groups in a double-blind trial, honey or sucrose (70 g in 250 mL of water per day) was given for 6 weeks. At the end of the study, the group that received honey demonstrated a decrease in TC, TG and LDL; HDL increased. However, the group that received sucrose had a decrease in HDL and an increase in TC, TG and LDL [39]. In this study, the lowest LDL was found in the low-dose stevia administration. This as well as the high dose was significantly lower than the control group. However, the reduction was not in a dose-dependent manner. However, the aqueous extract of stevia (*Stevia rebaudiana* Bertoni leaves) in another study lowered LDL (33.02 ± 4.79 to 22.77 ± 4.36 mg/dL) in albino rats in a dose-dependent manner (200–500 ppm/kg b.wt.) [40]. In a small study in India, LDL was lowered when stevia was administered (20 mL of aqueous extract/200 mL) to 20 women with hyperlipidaemia [41]. This effect was however seen in one subject. It should be noted that most of the studies examining the effect of stevia on health use the pure aqueous extract, whereas in this study a highly processed commercialised stevia product was used. In a 3-arm parallel, triple-blind, randomised study on 138 overweight subjects, a test beverage was spiked with sucralose, sucrose and stevia. After 60 days, no changes in LDL or oxidized LDL were observed [42].

The most atherogenic TG-rich lipoprotein particle is VLDL [43] which LDL is a hepatic synthesized molecule and functions as a vehicle for the mobilization of TG from the liver to the peripheral. Contrary to our results, the aqueous extract of *Stevia rebaudiana* Bertoni leaves is said to have lowered VLDL from 21.22 ± 5.79 to 19.33 ± 5.95 mg/dL in experimental rats at a dose of 500 ppm for 8 weeks [40]. Elnaga et al. [44] published a remarkable effect of stevia on VLDL on female rats fed 25–1000 mg/kg/day for 12 weeks. However, levels of stevia were far above the WHO recommended doses; hence, extrapolation of results to humans becomes unrealistic. Also, in this study, a refined stevia sweetener was used as compared with the pure aqueous extract.

The CRI-I sometimes referred to as the cardiac risk ratio (CRR) is an indication of build-up of plaques in the coronary arteries. On the other hand, cardiovascular risk is predicted by CRI II. Even though the individual lipid parameters are fairly predictive of CVDs, some studies suggest that with an apparently normal or moderately high lipid parameter, the CRI I (TC/HDL) and II (LDL/HDL) which examines the relationships between the individual markers are alternative diagnostic predictors of cardiovascular events [26, 45].

The worst CRI I was seen in WSHD and the best in HLD. In the case of honey, it is believed that the nonsugar part is what confers this gain onto honey [46]. Similarly in a study using diabetic rats, a significant reduction was seen in CRI-I (CRI = TC/HDL cholesterol), cardiovascular risk index, and AI using 1 or 2 g/kg b.wt. of Nigeria honey but not 3 g/kg b.wt [47]. For CRI II, the WSHD had the highest value, and the SHD had the lowest (0.491 vs. 0.231). Both BSLD and HLD had relatively low CRI-II. Polyphenols present in honey are said to be protective against CVDs. The two Castelli indices have a high correlation because much of the cholesterol resides in the LDL; therefore, LDL and TC work in tandem [48].

A more reliable, strong and dependable indicator of the risk of cardiometabolic diseases in the general population that is beginning to gain prominence is the AIP. For white sugar, the AIP was in the high-risk group for all doses. BSLD and HLD demonstrated moderate risk. A dose-dependent increase was observed for stevia with strong statistical differences at all levels (*p* = 0.0003) compared to the control group. Conventionally, the various individual components of the lipid profile have been used to try to diagnose CVDs due to atherosclerosis. The AIP which is a calculated value is said to be more predictive of dyslipidaemia and LDL-c particle size [49]. The AIP is a reflection of the pre- and antiatherogenic particles associated with the AIP and also correlates with the composition and size of lipoproteins [50]. When calculated values less than 0.11 are indicative of a low risk of CVD, between 0.11 and 0.24 is moderate risk, and values above 0.24 are very suggestive of high risk [49, 51]. The low and moderate use of honey is said to have reduced cardiovascular risk by lowering TC and TG with an observed HDL increase. On the contrary, white sugar (low to high) increased cardiovascular risk by increasing LDL (and TC/HDL) [52].

Normal rat cages were used for the study instead of metabolic cages. Individual metabolic cages would have allowed for the researcher to closely monitor each rat's food and water intake and the physical activity of the rats. This study recapitulates the evidence on the health effect of excessive intake of sweeteners over time.

## 5. Conclusion

Chronic and excessive intake of refined sugar and stevia are potential risk factors in weight gain and appetite stimulation. Findings from this study demonstrated that high intake of white sugar over a long period has the potential in reducing HDL-c and increasing atherogenicity. Stevia has the potential of causing not only long weight gain but also stimulating appetite and risk of atherogenicity.

## Figures and Tables

**Table 1:** Composition of normal chow.

Nutrient distribution		Feed mix	
Nutrients	%/100 g	Ingredients	Quantity g/100 g
Carbohydrates		Maize	30.0
Sugars	43.3%	Concentrate	20.0
Fibre	26.7%	Wheat bran	50.0
Proteins	18.8%		
Fats and oils	4.5%		
Mineral vitamin mix	6.7%		

**Table 2:** Experimental design and feed formulation.

Experimental groups	No. of rats	Feed composition	Dosage (g/mL)
Normal control (C)	6	Normal chow	1
White sugar low dose (WSL)	6	Normal chow + 5% white sugar	0.035
White sugar medium dose (WSM)	6	Normal chow + 10% white sugar	0.07
White sugar high dose (WSHD)	6	Normal chow + 15% white sugar	0.1
Brown sugar low dose (BSLD)	6	Normal chow + 5% brown sugar	0.036
Brown sugar medium dose (BSMD)	6	Normal chow + 10% brown sugar	0.072
Brown sugar high dose (BSHD)	6	Normal chow + 15% brown sugar	0.11
Honey low dose (HLD)	6	Normal chow + 5% honey	0.047
Honey medium dose (HMD)	6	Normal chow + 10% honey	0.094
Honey high dose (HHD)	6	Normal chow + 15% honey	0.14
Stevia low dose (SLD)	6	Normal chow + 5% stevia	0.007
Stevia medium dose (SMD)	6	Normal chow + 10% stevia	0.014
Stevia high dose (SHD)	6	Normal chow + 15% stevia	0.021

**Table 3:** Effect of sweeteners on triglyceride concentration.

TG (mmol/L)	Low dose	Medium dose	High dose	*p* value
Control	0.77 ± 0.04			
White sugar	1.20 ± 0.17^a^	1.16 ± 0.17^a^	1.09 ± 0.17	0.877
Brown sugar	0.67 ± 0.08	1.48 ± 0.31	1.13 ± 0.24	0.074
Honey	0.94 ± 0.27	2.12 ± 0.16^a^	1.56 ± 0.25	**0.009** ^**∗**^
Stevia	1.99 ± 0.07^a^	2.44 ± 0.09^a^	2.66 ± 0.37^a^	0.127
*p* value	**0.0001** ^**∗**^	**0.0008** ^**∗**^	**0.0014** ^**∗**^	

*Note:* Results are presented as means ± SEM, *n* = 6. The overall *p* value for honey is 0.009, demonstrating a statistically significant effect on TG across different doses of honey. The *p* value for the high-dose group is 0.0014. This indicates a significant difference in TG among sweeteners at high doses. The *p* value for the medium-dose group is 0.0008. This indicates a significant difference in TG among sweeteners at medium doses. The *p* value for the low-dose group is 0.0001. This indicates a significant difference in TG among sweeteners at low doses.

^∗^Values are statistically significant at *p* < 0.05.

^a^Values are significantly different from the control.

**Table 4:** Effect of sweeteners on HDL-c concentration.

HDL-c (mmol/L)	Low dose	Medium dose	High dose	*p* value
Control		1.29 ± 0.17		
White sugar	1.33 ± 0.11	1.34 ± 0.12	1.02 ± 0.16	0.332
Brown sugar	1.45 ± 0.12	1.54 ± 0.08	1.42 ± 0.07	0.648
Honey	1.72 ± 0.19	1.42 ± 0.05	1.43 ± 0.08	0.169
Stevia	1.43 ± 0.11	1.54 ± 0.09	1.56 ± 0.09	0.594
*p* value	0.228	0.351	**0.008** ^**∗**^	

*Note:* Results are presented as means ± SEM, *n* = 6. The *p* value for the high-dose group is 0.008, indicating a significant difference in HDL-c among sweeteners at high doses.

^∗^Values are statistically significant at *p* < 0.05.

**Table 5:** Effect of sweeteners on LDL-c concentration.

LDL-c (mmol/L)	Low dose	Medium dose	High dose	*p* value
Control		0.52 ± 0.051		
White sugar	0.48 ± 0.05	0.55 ± 0.07	0.44 ± 0.04	0.374
Brown sugar	0.41 ± 0.04	0.49 ± 0.02	0.36 ± 0.03^a^	0.043
Honey	0.44 ± 0.01	0.36 ± 0.03^a^	0.37 ± 0.03^a^	0.084
Stevia	0.35 ± 0.04^a^	0.39 ± 0.03	0.36 ± 0.03^a^	0.567
*p* value	0.139	**0.017** ^**∗**^	0.299	

*Note:* Results are presented as means ± SEM, *n* = 6. The *p* value for the medium-dose group is 0.017. This indicates a significant difference in LDL-c among sweeteners at medium doses.

^∗^Values are statistically significant at *p* < 0.05.

^a^Values are significantly different from the control.

**Table 6:** Effect of sweeteners on VLDL-c concentration.

VLDL-c (mmol/L)	Low dose	Medium dose	High dose	*p* value
Control		0.18 ± 0.02		
White sugar	0.25 ± 0.03	0.23 ± 0.03	0.23 ± 0.03	0.922
Brown sugar	0.18 ± 0.02	0.52 ± 0.26	0.68 ± 0.24^a^	0.247
Honey	0.18 ± 0.05	0.45 ± 0.03	0.32 ± 0.05	**0.005** ^**∗**^
Stevia	0.38 ± 0.02	0.48 ± 0.02	0.53 ± 0.07	0.056
*p* value	**0.001** ^**∗**^	0.443	**0.016** ^**∗**^	

*Note:* Results are presented as means ± SEM, *n* = 6. The overall *p* value for honey is 0.005, demonstrating a statistically significant effect on VLDL across different doses of honey. The *p* value for the high-dose group is 0.016. This indicates a significant difference in VLDL among sweeteners at high doses. The *p* value for the low-dose group is 0.001. This indicates a significant difference in VLDL among sweeteners at low doses.

^∗^Values are statistically significant at *p* < 0.05.

^a^Values are significantly different from the control.

**Table 7:** Effect of sweeteners on Castelli Risk Index I.

CRI-I	Low dose	Medium dose	High dose	*p* value
Control	1.86 ± 0.12			
White sugar	1.74 ± 0.19	1.79 ± 0.11	2.30 ± 0.36	0.229
Brown sugar	1.51 ± 0.06	1.55 ± 0.05	1.39 ± 0.09	0.219
Honey	1.36 ± 0.09	1.37 ± 0.08	1.44 ± 0.03	0.724
Stevia	1.43 ± 0.02	1.40 ± 0.03	1.39 ± 0.02	0.478
*p* value	0.113	**0.0009** ^**∗**^	**0.005** ^**∗**^	

*Note:* Results are presented as means ± SEM, *n* = 6. The *p* value for the high-dose group is 0.005. This indicates a significant difference in CRI-I among sweeteners at high doses. The *p* value for the medium-dose group is 0.0009. This indicates a significant difference in CRI-I among sweeteners at medium doses.

^∗^Values are statistically significant at *p* < 0.05.

**Table 8:** Effect of sweeteners on Castelli Risk Index II.

CRI-II	Low dose	Medium dose	High dose	*p* value
Control	0.42 ± 0.02			
White sugar	0.37 ± 0.05	0.40 ± 0.02	0.49 ± 0.09	0.369
Brown sugar	0.29 ± 0.03	0.32 ± 0.02	0.25 ± 0.02^a^	0.178
Honey	0.26 ± 0.02^a^	0.25 ± 0.03^a^	0.26 ± 0.02^a^	0.941
Stevia	0.24 ± 0.01^a^	0.26 ± 0.02^a^	0.23 ± 0.01^a^	0.371
*p* value	0.052	**0.0001** ^**∗**^	**0.0024** ^**∗**^	

*Note:* Results are presented as means ± SEM, *n* = 6. The *p* value for the medium-dose group is 0.0001, indicating a significant difference in CRI-II among sweeteners at medium doses. The *p* value for the high-dose group is 0.0024. This indicates a significant difference in CRI-II among sweeteners at high doses.

^∗^Values are statistically significant at *p* < 0.05.

^a^Values are significantly different from the control.

**Table 9:** Effect of sweeteners on the Atherogenic Index of Plasma.

AIP	Low dose	Medium dose	High dose	*p* value
Control	0.18 ± 0.04			
White sugar	0.39 ± 0.07	0.33 ± 0.05	0.38 ± 0.08	0.349
Brown sugar	0.20 ± 0.08	0.52 ± 0.18	0.46 ± 0.11	0.127
Honey	0.25 ± 0.12	0.78 ± 0.05^a^	0.61 ± 0.06	**0.002** ^**∗**^
Stevia	0.77 ± 0.05^a^	0.79 ± 0.04^a^	0.85 ± 0.06^a^	0.6105
*p* value	**0.0003** ^**∗**^	0.196	**0.018** ^**∗**^	

*Note:* Results are presented as means ± SEM, *n* = 6. The overall *p* value for honey is 0.002, demonstrating a statistically significant effect on AIP across different doses of honey. The *p* value for the high-dose group is 0.018. Indicating a significant difference in AIP among sweeteners at high doses. The *p* value for the low-dose group is 0.0003. Indicating a significant difference in AIP among sweeteners at low doses.

^∗^Values are statistically significant at *p* < 0.05.

^a^Values are significantly different from the control.

## Data Availability

The data that support the findings of this study are available from the corresponding author upon reasonable request.
